# Ultrasound-guided disc pain induction test for diagnosis of discogenic lumbar pain: a cross-sectional study

**DOI:** 10.1186/s13018-023-04327-x

**Published:** 2023-11-08

**Authors:** Keisuke Masuda, Hideki Shigematsu, Manabu Maeda, Akinori Okuda, Yasuhito Tanaka

**Affiliations:** 1 Department of Emergency and Critical Care Medicine, Nara Medical University, 840 Shijo-cho, Kashihara City, Nara 6348522 Japan; 2 Department of Orthopedic Surgery, Nara Medical University, 840 Shijo-cho, Kashihara City, Nara 6348522 Japan; 3 Department of Orthopedic Surgery, Maeda Orthopaedic Clinic, 864-1, Kideracho, Nara City, Nara 6308306 Japan

**Keywords:** Lower back pain, Discogenic pain, Discoblock, Lumbar pain, Disc degeneration

## Abstract

**Background:**

Several methods can be used to diagnose discogenic pain, but only discoblock can diagnose discogenic pain definitively. This study aimed to examine the usefulness of an ultrasound-guided disc pain induction test for a simple and accurate diagnosis of the culprit lesion.

**Methods:**

We included 41 patients with lumbar pain in whom pain was induced by an ultrasound-guided disc pain induction test. All patients had confirmed pain at L1/2 to L5/S1 based on an ultrasound-guided disc pain induction test and underwent X-ray photography and magnetic resonance imaging. Seventeen patients who required injection due to severe pain underwent discoblock procedures for discs with the most intense pain, and visual analogue scale (VAS) scores were obtained before and after the procedure for these patients. We analysed the association between painful discs and radiological findings.

**Results:**

Pain induction was noted in a total of 65 discs, and the pain was induced in 23 patients in only one disc. All patients had disc degeneration of Pfirrmann classification grade 1 or higher, with more significant disc degeneration in painful discs than in painless discs. There was no significant relationship between the presence or absence of pain and Modic type. The average VAS measurements improved significantly from 9.5 (pre-procedure) to 2.5 (post-procedure). These results suggest that the most painful discs were the causes of discogenic lumbar pain.

**Conclusions:**

Our ultrasound-guided disc pain induction test may help diagnose disc degeneration and identify culprit lesions, even when multiple discs exhibit findings of degeneration.

**Supplementary Information:**

The online version contains supplementary material available at 10.1186/s13018-023-04327-x.

## Background

Lower back pain is a very common symptom, with a reported lifetime prevalence of 84% [1]. Previously, non-specific lower back pain accounted for 85% of cases; however, improvements in diagnostic approaches have led to more than 75% of cases being diagnosed pathologically and anatomically, with non-specific lower back pain now accounting for only approximately 22% of cases [2]. Identifying the specific causes of lower back pain enables practitioners to treat each cause with an appropriate method.

Research suggests that discogenic lumbar pain accounts for 13% of all cases of lower back pain [2]. Discogenic lumbar pain is typically diagnosed based on physical manifestations, such as lumbar pain at anterior bending and deep pain, as well as magnetic resonance imaging (MRI) findings. However, a definitive diagnosis is difficult to make when images suggest abnormal findings in multiple discs, thus requiring discography and discoblock procedures to confirm a diagnosis [2–4].

Recent advancements in ultrasound have led to improvements in the diagnosis of orthopaedic diseases, including those affecting the spine [5, 6]. Among them, palpation combined with ultrasonography (i.e., sonopalpation) allows for accurate palpation even at deeper structures [7, 8]. Our group has developed a novel ultrasound-guided disc pain induction test that relies on sonopalpation to detect discogenic lumbar pain. In this test, manual pressure is applied to each disc during real-time ultrasound monitoring at the disc level from the ventral side. In the present study, we aimed to examine the usefulness of this method for the simple and accurate diagnosis of culprit lesion(s) in patients with discogenic lumbar pain.

## Methods

### Patients and assessments

This cross-sectional study was approved by the ethics committee of Higashiosaka City Medical Center (approval number: 02-0760-A), and written or opt-out informed consent was obtained from all participants.

The study included 41 patients who had visited the outpatient ward of the Department of Orthopedics at our hospital for chief concerns of lower back pain between January 2019 and January 2022. The inclusion criterion was that pain was confirmed based on the results of an ultrasound-guided disc pain induction test. The exclusion criterion was a history of lumbar spine surgery. Patients were assessed for increases in pain during anterior–posterior bending of the trunk and pain induced between L1/2 and L5/S1 during ultrasonography. Discs in which the pain was induced were defined as the “painful disc”. When pain could be induced in more than one disc, the disc in which the most intense pain was induced was defined as the “most painful disc”. A PLT-1005BT linear probe (3.8–14.0 MHz), a PVI-475BX convex probe (1.8–6.4 MHz) (AplioMX and Aplio i800 systems; Canon Medical System Company, Tochigi, Japan), or a C1-5-RS convex probe (2.0–5.5 MHz) (LOGIQ e Premium; GE healthcare, Chicago, USA) was used for ultrasonography. All patients underwent X-ray photography (XP) and MRI of the lumbar spine, in which disc degeneration was graded based on the Pfirrmann classification [9]. Modic types were also assessed at each vertebral body [10]. MR images and X-ray photographs were also examined for abnormalities, such as disc herniation and high-intensity zones [11]. Patients who required injection due to severe pain underwent discoblock procedures for the most painful disc.

Visual analogue scale (VAS) scores were obtained before and after the procedure for patients who had chosen to undergo discoblock to assess pain response. Pfirrmann classifications and Modic types were compared between painful discs and painless discs, as well as between the most painful discs and other discs. The pre-procedure and post-procedure VAS results were compared to evaluate the effect of the discoblock procedure.

### Procedure for the ultrasound-guided disc pain induction test

**Fig. 1:**
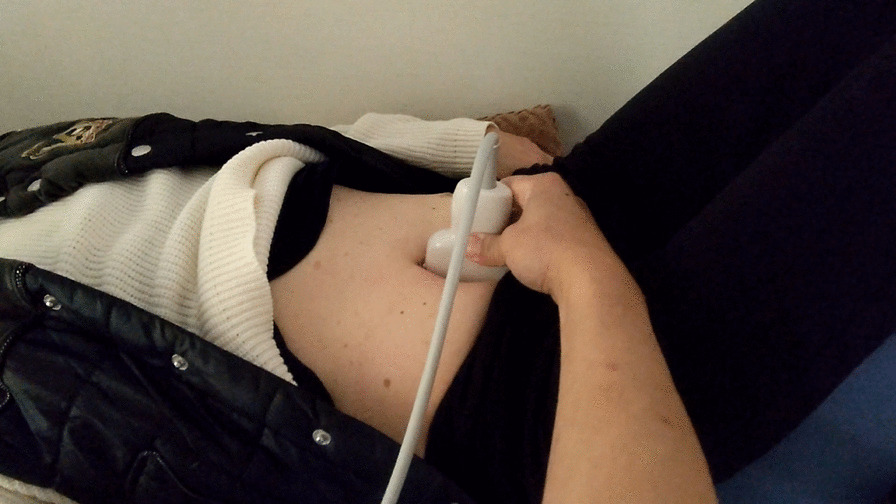
Photograph showing the ultrasound-guided disc pain induction test process. The long axis of the spine is visualized by placing the patient in the supine position with both knees flexed and applying the ultrasound probe on the patient’s ventral side

**Fig. 2:**
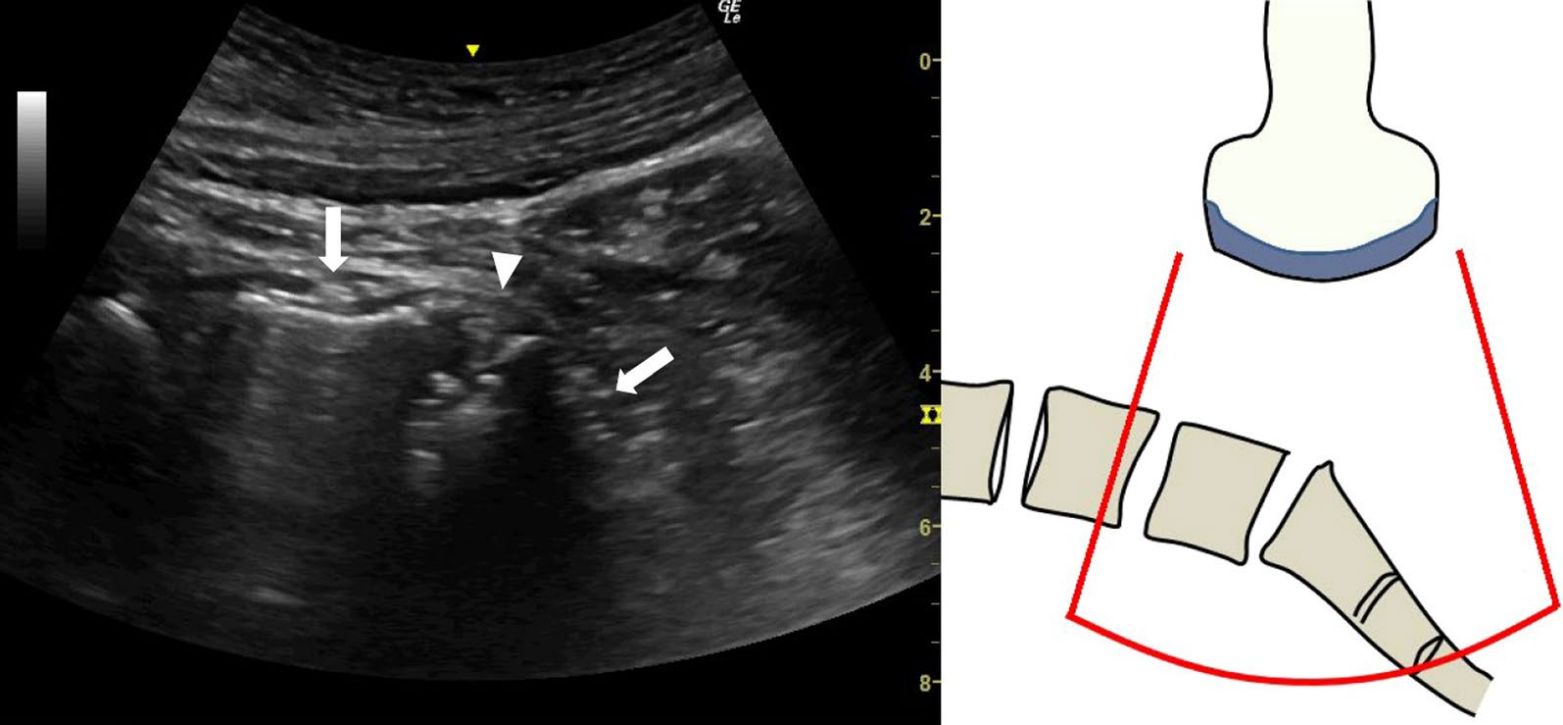
Long-axis image of the lumbar spine showing the L5/S1 intervertebral disc (arrowhead) and the outline of the L5 and S1 vertebral bodies (arrow). The outline of the S1 vertebral body has a characteristic shape

The patient was placed in the supine position with both knees flexed, following which the ultrasound probe was placed on the patient’s ventral side to visualize the long axis of the spine (Fig. 1). Although intestinal gas usually obstructs spinal visualization, pressure is applied using the ultrasound probe to remove intestinal gas from the view and allow for visualization of the intervertebral discs. Hyperechoic visualization of the disc contour thus allows the examiner to determine whether there is bulging of discs between the vertebral bodies (Fig. 2).

**Fig. 3:**
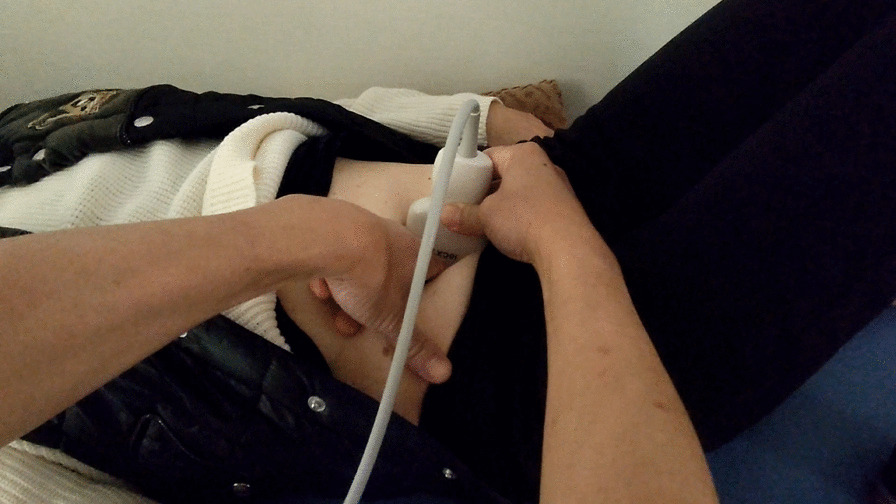
Photograph showing the ultrasound-guided disc pain induction test process. The examiner inserts a finger between the probe and the disc to palpate the target disc while compressing the site to check for induced pain

**Fig. 4:**
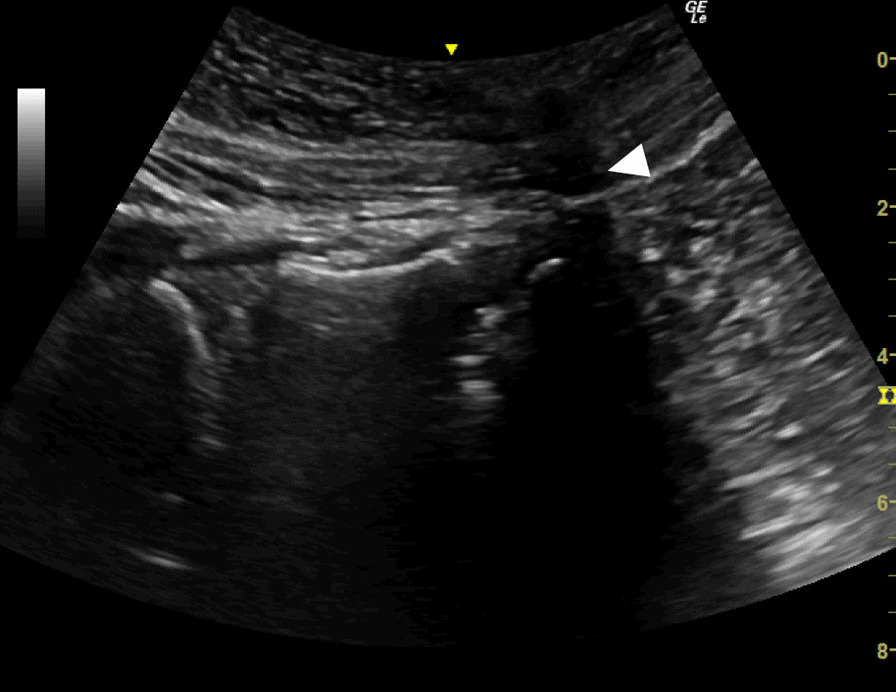
Long-axis image of the lumbar spine during manual application of pressure at the L5/S1 intervertebral disc. The arrowhead shows an acoustic shadow directly above the L5/S1 disc level

The disc level is confirmed by identifying the L5/S1 disc, which has a characteristic vertebral contour, and the probe is moved such that the target disc is centred on the display. The examiner then inserts a finger between the probe and the disc to palpate the target disc while compressing the site to check for induced pain (Fig. 3). The acoustic shadow created by the finger is used to confirm that the examiner’s finger is located over the target disc (Fig. 4) (Additional file 1: Video S1).

**Fig. 5:**
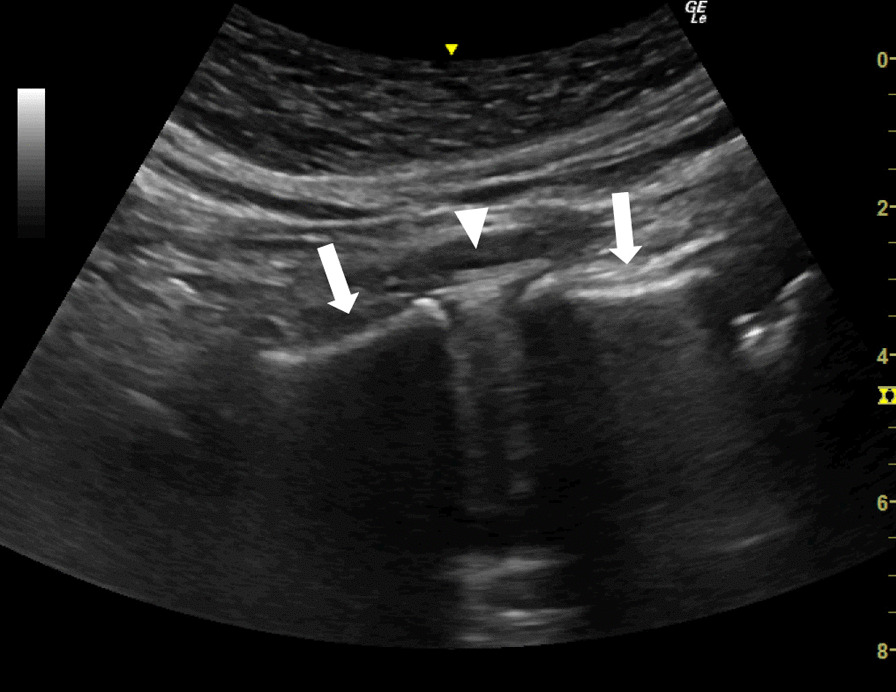
Long-axis image of the lumbar spine after moving the probe cranially showing the L4/L5 intervertebral disc (arrowhead) and the outline of the L4 and L5 vertebral bodies (arrow)

After one disc is compressed, the probe is moved cranially, and pressure is sequentially applied to each disc to check for induced pain from L1/2 to L5/S1 (Fig. 5) (Additional file 2: Video S2).

### Statistical analysis

The Kruskal–Wallis test was used to compare the Pfirrmann classification between painful discs and painless discs, and the Steel–Dwass multiple comparison tests were used for post hoc analysis. Cramer’s association coefficient (*V*) was used to assess the association between Modic types and painful discs, with a *V* value closer to 1 signifying a greater association. The Wilcoxon signed-rank test was used to assess the pain response to the discoblock procedure. The level of statistical significance was set at *p* < 0.05. Statistical analyses were performed using EZR (Saitama Medical Center, Jichi Medical University, Saitama, Japan).

## Results

Pain was confirmed in 44 patients based on the results of an ultrasound-guided disc pain induction test. Three patients were excluded, and the remaining 41 patients were included for further analysis. Demographic data and examination findings for the 41 included patients (11 men and 30 women) are summarized in Table 1. The median age was 43.5 (range 28.0–60.5) years. Pain during anterior bending was the most common symptom, affecting 25 patients (61%). Anterior–posterior bending did not increase pain in five patients (12%). Pain only during posterior bending was observed in six patients (15%). Pain during both anterior and posterior bending was observed in five patients (12%). XP and MRI findings showed disc herniation in 17 patients (41%), high-intensity zone in 17 patients (41%), degenerative spondylolisthesis in one patient (2%), and spondylolytic spondylolisthesis in one patient (2%).

**Table 1:** Demographic data and findings of examination

Demographic data	
Number of patients (M:F)	41 (11:30)
Age (years)	43.5 (28.0–60.5)
Pain at anterior–posterior bending of the trunk (*n* = 41)	
Anterior bending	25
Posterior bending	6
Both of anterior and posterior bending	5
None	5
MRI findings	
Disc herniation	17
High intensity zone	17
Degenerative spondylolisthesis	1
Spondylolytic spondylolisthesis	1

Table 2 summarizes the results of the ultrasound-guided disc pain induction test, which identified pain in a total of 65 discs. Disc tenderness was most common at L4/5, affecting 31 patients (48%), and tenderness in a single disc was the most common (23 patients, 56%).

**Table 2:** Results of ultrasonographic-guided disc pain induction test

Location of tender disc (*n* = 65)	
L1/2	0
L2/3	3
L3/4	13
L4/5	31
L5/S1	18
Number of tender disc (*n* = 41)	
1	23
2	12
3	6

Table 3 summarizes the comparison between painless and painful discs based on MRI findings. All painful discs had Pfirrmann classifications of grade 1 or higher and showed signs of more advanced degeneration compared with painless discs (*p* < 0.05). There was no clear association between the presence or absence of induced pain and Modic type (*V* = 0.18).

**Table 3:** Comparison between painless disc and induced pain disc about MRI findings

Pfirrmann classification	Painless disc (*n* = 140)	Induced pain disc (*n* = 65)	*p* value
0	108	0	*p* < 0.05
1	0	5	
2	1	5	
3	9	19	
4	19	33	
5	3	3	

Modic type	Painless disc (*n* = 140)	Induced pain disc (*n* = 65)	Cramer's *V*
0	132	56	*V* = 0.18
1	1	4	
2	6	4	
3	1	1	

Table 4 summarizes comparisons among painful, painless, and other painful discs based on MRI findings. Discogenic pain was associated with more advanced signs of degeneration compared with painless discs (*p* < 0.05). However, there was no significant difference in the degree of disc degeneration between the most painful disc and other painful discs. There was no clear association between the Modic type and any of the three types of discs (*V* = 0.23).

**Table 4:** Comparison between most painful disc, other pain disc, and painless disc about MRI findings

Pfirrmann classification	Painless disc (*n* = 140)	Other painful disc (*n* = 24)	Most painful disc (*n* = 41)	*p* value
0	108	0	0	*p* < 0.05
1	0	4	1	
2	1	0	5	
3	9	9	10	
4	19	11	22	
5	3	0	3	

Modic classification	Painless disc (*n* = 140)	Other painful disc (*n* = 24)	Most painful disc (*n* = 41)	Cramer's *V*
0	132	23	33	*V* = 0.23
1	1	1	3	
2	6	0	4	
3	1	0	1	

Seventeen patients who required injection due to severe pain underwent discoblock procedures for the most painful disc. VAS scores improved by three or more points in 16 out of 17 patients. Overall, significant improvements in pain levels were observed, with the pre-procedure and post-procedure VAS scores being 9.5 ± 0.9 and 2.5 ± 2.7, respectively. Based on these results, the positive predictive value of the ultrasound-guided disc pain induction test was 94%.

## Discussion

The current results suggest that our ultrasound-guided disc pain induction test can be used for simple, non-invasive identification of the culprit lesion(s) in patients with discogenic lumbar pain. Painful discs exhibited significant disc degeneration compared with painless discs. These findings are in accordance with those of previous studies reporting associations of disc protrusion and disc degeneration on MRI with lumbar pain [3, 12]. However, we observed no association between tenderness and Modic type. Although studies have suggested that Modic type is associated with disc degeneration [10], which is often observed in patients with lower back pain [3], Modic changes in the vertebral body occur due to spinal degeneration and are not directly associated with discogenic lumbar pain.

A high-intensity zone on MRI is a characteristic finding in patients with discogenic lumbar pain; however, this finding was not observed in more than half of the patients in the current study. Some reports suggest that the presence of a high-intensity zone is not sufficient for the accurate identification of the cause or level of pain [12], as high-intensity zones can be observed even in asymptomatic patients [13]. Therefore, concurrent sonopalpation may be ideal for improving diagnostic accuracy.

In this study, discoblock procedures at the disc with the most intense pain were effective, indicating that the disc in which the most intense pain is induced during the ultrasound-guided disc pain induction test is likely the cause of discogenic lumbar pain. This result supports the value of our method for improving diagnostic accuracy for discogenic lumbar pain. The discography and discoblock procedure are useful for the definitive diagnosis of discogenic pain in the lumbar spine. A previous report described that pain relief following an injection of a small amount of local anaesthetic agent into the painful disc was a useful tool for the diagnosis of discogenic low back pain compared with discography [4]. A review into the indication for discography has revealed the following [14]:

1. Further evaluation of demonstrable abnormal discs to help assess the extent of abnormality or correlation of the abnormality with the clinical symptoms. Such symptoms may include recurrent pain from a previously operated disc or due to lateral disc herniation.
2. Patients with persistent, severe symptoms where other diagnostic tests have failed to reveal clear confirmation of a suspected disc as the source of pain.
3. Assessment of patients who have failed to respond to surgical intervention to determine if there is painful pseudarthrosis or a symptomatic disc in a posteriorly fused segment. The aim is to evaluate the risk of recurrent disc herniation.
4. Assessment of discs before fusion to determine if the discs within the proposed fusion segment are symptomatic and to determine if the discs adjacent to this segment are normal.
5. Assessment of candidates for minimally invasive surgical intervention to confirm a contained disc herniation or to investigate the dye distribution pattern prior to chemonucleolysis or percutaneous procedures.

The review also discussed the complications of discography such as spinal headaches, discitis, meningitis, intrathecal haemorrhage, arachnoiditis, reaction to accidental intradural injection and damage to the disc itself. However, these complications were mainly reported from the late 1940s to the mid-1960s. The most frequent and severe complication now reported from discography is discitis, and the overall incidence calculated by adding all the occurrences together was less than 0.25% by patient and less than 0.14% by disc. The review described that the improvement of radiographic equipment, improved technique and smaller needles have made the procedure increasingly safer. However, it is still important to avoid unnecessary testing. Given that our ultrasound-guided disc pain induction test is simple and non-invasive, it may aid in the early diagnosis of the culprit lesion in patients with discogenic lumbar pain, thereby reducing unnecessary discography procedures.

In the present study, disc pain was most common at L4/5, and however, we could not identify past reports that have described the distribution of discogenic low back pain. Since a past population-based cohort study in our country showed the prevalence of lumbar disc degeneration was highest at L4/5 [15], this is considered to be consistent with our research results. However, there are reports that low back pain that presents in a similar manner to discogenic low back pain is associated not only with disc degeneration but also with endplate signal changes and Schmorl nodules. Therefore, it is difficult to diagnose discogenic low back pain based solely on the presence of disc degeneration [16]. We believe that clinical symptoms along with imaging finding are important in the diagnosis of discogenic low back pain.

In the present study, characteristic pain during anterior bending of the trunk was observed in 61% of patients, while 12% of patients experienced no increase in pain intensity during anterior–posterior bending of the trunk. A previous report has described that in discogenic low back pain patients, sensitivity was 0.650 and specificity was 0.311 for the restriction of lumbar flexion range of motion, whereas sensitivity was 0.175 and specificity was 0.636 for the restriction of lumbar extension range of motion [2]. The report suggests that several examinations should be conducted to confirm the final diagnosis in low back pain. The axis of rotation in the spine during anterior–posterior bending of the trunk is reportedly located in the posterior disc region [17–20], suggesting that disc pressure increases during posterior bending. Many patients do not experience such characteristic mechanical pain, resulting in a diagnosis of non-specific lumbar pain.

Previous studies have indicated that discogenic lumbar pain results from vascular ingrowth into the inner lining of the cartilage endplates and annulus fibrosus, along with nerve ingrowth into the inner annulus fibrosus and nucleus pulposus of the injured disc [21, 22]. Discogenic pain may, therefore, occur due to increases in disc pressure during anterior bending, which stimulates the ingrown nerve. Effective sonopalpation methods for diagnosing discogenic pain in the cervical spine have been documented [7]. Furthermore, as pressure is applied to the discs at each level in real-time, the ultrasound-guided disc pain induction test may help to identify the culprit lesion(s) by increasing the internal pressure in the individual discs. Diagnosis based on provocative testing under ultrasound guidance is primarily advantageous given the ability to avoid the use of iodine-based contrast agents in patients with allergies, as well as the exposure of both patients and examiners to ionizing radiation.

This study had some limitations, including possible selection bias, as well as unclear sensitivity and specificity of the test. Furthermore, not all patients underwent discoblock procedures, and we did not examine the influence of patient physique or disc level on the ability to ensure proper application of pressure.

## Conclusions

Despite the study limitations, our results suggest that the disc bearing the most intense pain in the ultrasound-guided disc pain induction test can be identified as the culprit lesion in patients with discogenic lumbar pain. In addition, our results indicate that disc pain was significantly associated with the degree of disc degeneration. Thus, the ultrasound-guided disc pain induction test represents a simple, non-invasive method for the diagnosis of discogenic lumbar pain. Further studies, such as multicentre studies and studies verifying the accuracy of the ultrasound-guided disc pain induction test in patients with discogenic low back pain, are required.

### Supplementary Information

**Additional file 1: Video S1.** Long-axis image of the lumbar spine during manual application of pressure at the L5/S1 intervertebral disc.

**Additional file 1: Video S2.** Long-axis image of the lumbar spine during manual application of pressure at the L4/L5 intervertebral disc.

## Data Availability

Not applicable.

## Abbreviations

XP: X-ray photography
MRI: Magnetic resonance imaging
VAS: Visual analogue scale
